# A genome-wide single nucleotide polymorphism and copy number variation analysis for number of piglets born alive

**DOI:** 10.1186/s12864-019-5687-0

**Published:** 2019-04-27

**Authors:** Nedenia Bonvino Stafuzza, Rafael Medeiros de Oliveira Silva, Breno de Oliveira Fragomeni, Yutaka Masuda, Yijian Huang, Kent Gray, Daniela A. Lino Lourenco

**Affiliations:** 1 Department of Exact Science, School of Agricultural and Veterinarian Sciences (FCAV), Sao Paulo State University (UNESP), Jaboticabal, SP 14884-900 Brazil; 2 Department of Animal and Dairy Science, University of Georgia, Athens, GA USA; 3 National Center for Cool and Cold Water Aquaculture (NCCCWA), Agricultural Research Service, United States Department of Agriculture, Kearneysville, WV USA; 4 Department of Animal Science, University of Connecticut, Storrs-Mansfield, CT USA; 5 Smithfield Premium Genetics Group, Rose Hill, NC USA

**Keywords:** CNV, Complex trait, GWAS, SNP panel, *Sus scrofa domesticus*

## Abstract

**Background:**

In this study we integrated the CNV (copy number variation) and WssGWAS (weighted single-step approach for genome-wide association) analyses to increase the knowledge about number of piglets born alive, an economically important reproductive trait with significant impact on production efficiency of pigs.

**Results:**

A total of 3892 samples were genotyped with the Porcine SNP80 BeadChip. After quality control, a total of 57,962 high-quality SNPs from 3520 Duroc pigs were retained. The PennCNV algorithm identified 46,118 CNVs, which were aggregated by overlapping in 425 CNV regions (CNVRs) ranging from 2.5 Kb to 9718.4 Kb and covering 197 Mb (~ 7.01%) of the pig autosomal genome. The WssGWAS identified 16 genomic regions explaining more than 1% of the additive genetic variance for number of piglets born alive. The overlap between CNVR and WssGWAS analyses identified common regions on SSC2 (4.2–5.2 Mb), SSC3 (3.9–4.9 Mb), SSC12 (56.6–57.6 Mb), and SSC17 (17.3–18.3 Mb). Those regions are known for harboring important causative variants for pig reproductive traits based on their crucial functions in fertilization, development of gametes and embryos. Functional analysis by the Panther software identified 13 gene ontology biological processes significantly represented in this study such as reproduction, developmental process, cellular component organization or biogenesis, and immune system process, which plays relevant roles in swine reproductive traits.

**Conclusion:**

Our research helps to improve the understanding of the genetic architecture of number of piglets born alive, given that the combination of GWAS and CNV analyses allows for a more efficient identification of the genomic regions and biological processes associated with this trait in Duroc pigs. Pig breeding programs could potentially benefit from a more accurate discovery of important genomic regions.

**Electronic supplementary material:**

The online version of this article (10.1186/s12864-019-5687-0) contains supplementary material, which is available to authorized users.

## Background

Reproductive performance in pig production systems is usually quantified by several economically important traits. The number of piglets born alive is an important trait in pig breeding programs due to its significant impact on the production efficiency; however, this is a difficult trait to improve because of low prediction accuracy and heritability [1–3]. Although the heritability of reproductive traits is moderate to low, genetic improvements can be obtained using genomic tools to explore the chromosomal regions and genes that explain the variation in reproductive traits. In addition to that, genomic acts as an extra source of information increasing prediction accuracy even for lowly heritable traits [4].

Until now, a total of 27,465 quantitative trait loci (QTL) have been mapped in the porcine genome for 663 different traits (Pig QTLdb Release 35). From about 2058 QTL described for reproduction traits, a total of 1004 QTL were described for litter traits, of which 163 QTL are related to number of piglets born alive [5]. Studies have reported the association of several genes and genomic regions with number of piglets born alive in several pig breeds [1–3, 6–10]. Genome-wide association studies (GWAS) became very powerful tools to investigate genetic architecture of economically important traits in livestock, allowing detection of genomic regions and genes controlling polygenic traits related to reproductive performance in pigs [2, 3, 8].

The combination of GWAS with other genomic approaches can provide a better understanding about genes and pathways involved in complex traits [11]. In several livestock species, it has been demonstrated useful to integrate GWAS and copy number variation (CNV) analysis to advance the knowledge of economically important complex traits [12, 13].

Structural variants such as CNV represent an important source of genomic variation in mammalian genomes. They can be defined as segments of DNA (conventionally > 1 Kb) that differ in number of copies compared to a reference genome [14]. Compared with SNPs, CNVs cover wider chromosomal regions and may potentially be responsible for changes in gene structure, modifications in gene regulation, changes in gene dosage, and exposing recessive alleles, resulting in large phenotypic effects. A well-characterized phenotypic variation affected by CNV in pig is the white coat phenotype generated by the duplication of the KIT gene [15].

Although several methodologies have been applied to detect CNVs in the pig genome, SNP arrays have better properties, such as lower cost compared to next generation sequencing (NGS) methodology and can be used for both GWAS and CNV detections. Employing SNP for CNV detection, previous studies have identified thousands of porcine CNVs [12, 16–28]. Because most of these studies comprised relatively small numbers of animals, the impacts of CNVs on phenotypes are still relatively poorly understood.

In animal breeding and genetics, the commercial populations are usually large and comprise phenotypes, pedigree, and genotypes for a fraction of pedigreed animals. Single-step genomic best linear unbiased prediction (ssGBLUP) was developed to predict breeding values when this type of data is available [29]. This is the method of choice for such populations because traditional GWAS methods cannot be implemented directly due to the necessity of combining results with pedigree structure to create pseudo-observations [30, 31]. When the structure of the genotyped dataset is complex, problems such as double counting of contributions from pedigree and phenotypes, and preselection bias [32] reduce accuracy. Lately, ssGBLUP was extended to GWAS [33, 34]. The GWAS under the ssGBLUP framework is called ssGWAS and allows the combination of phenotypes, pedigree and genotypes in one single analysis with no need of calculating pseudo-observations [35, 36]. In ssGBLUP, the main assumption is that all SNP explain the same proportion of additive genetic variance; however, this is not biologically true, especially when the traits are affected by large QTL. To account for the fact SNP may explain different proportion of variance, weighted ssGBLUP can be used. The WssGBLUP (weighted single-step approach for genome-wide association) method weighs SNP according to their effects in an iterative way [33]. The WssGWAS is fast, accurate and simple to implement for genome-wide association studies [33].

The aim of this study was to perform a WssGWAS to effectively identify genomic regions and biological processes related to number of piglets born alive in the Duroc breed and perform a CNV analyses to detect potential regions affecting the phenotype through changes in gene dosage. The elucidation of genes and molecular mechanisms controlling this trait should result in a better understanding of the genetic regulation of reproductive performance.

## Results

### CNV detection

After applying stringent filtering criteria, 653 samples did not pass the filtering and were discarded. The 2867 remaining samples were explored to search for CNV.

A total of 46,118 CNVs (4892 gains and 41,226 losses) were detected by PennCNV, of which 8152 (645 gain and 7507 loss) were non-redundant CNVs among the total of 2865 samples. CNVs were not identified in two samples. All CNVs detected by PennCNV were used to infer CNVR by aggregating overlapping CNVs. Thus, a total of 425 CNVRs were obtained (Additional file 1).

**Fig. 1:**
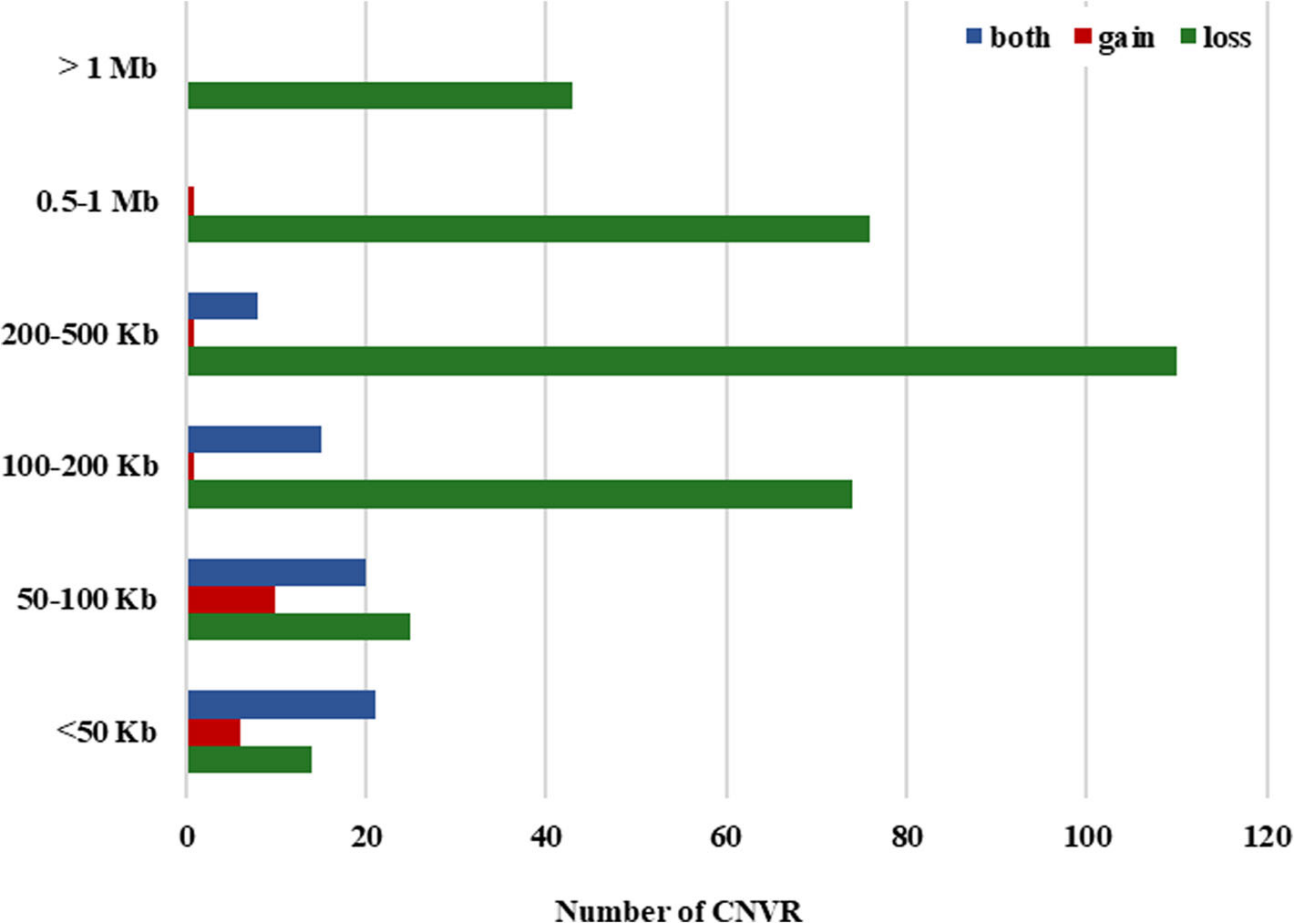
The number of CNVRs identified in this study according to the size interval and state (gain, loss or both)

The size of the CNVRs averaged 463,621 bp and ranged from 2552 bp to 9718,410 bp (Fig. 1). Among these regions, 342 corresponded to copy losses, 19 to copy gains and 64 to both (the same fragment showed losses or gains in different animals). It corresponds to a loss:gain ratio of 4.89.

The CNVRs inferred in our study covered 197,038,894 bp (7.01%) of the autosomal genome sequence, and their frequencies ranged from 0.5 to 53.61% in this Duroc population (Table 1).

**Table 1:** Chromosome distribution of all 425 CNVRs detected in the porcine genome

Chr	Chr length (bp)	CNVR number	Length of CNVR (bp)	%
SSC1	315,321,322	49	12,420,297	3.94
SSC2	162,569,375	33	17,317,379	10.65
SSC3	144,787,322	30	12,626,965	8.72
SSC4	143,465,943	15	10,565,509	7.36
SSC5	111,506,441	17	9,232,945	8.28
SSC6	157,765,593	22	12,493,722	7.92
SSC7	134,764,511	26	10,659,114	7.91
SSC8	148,491,826	36	10,789,394	7.26
SSC9	153,670,197	19	12,793,931	8.32
SSC10	79,102,373	16	8,515,041	10.76
SSC11	87,690,581	22	14,058,242	16.03
SSC12	63,588,571	25	8,687,209	13.66
SSC13	218,635,234	24	11,779,507	5.38
SSC14	153,851,969	27	9,624,904	6.25
SSC15	157,681,621	26	23,006,893	14.59
SSC16	86,898,991	12	5,426,107	6.24
SSC17	69,701,581	10	3,519,539	5.05
SSC18	61,220,071	16	3,522,196	5.75
Total	2,808,509,378	425	197,038,894	7.01

**Fig. 2:**
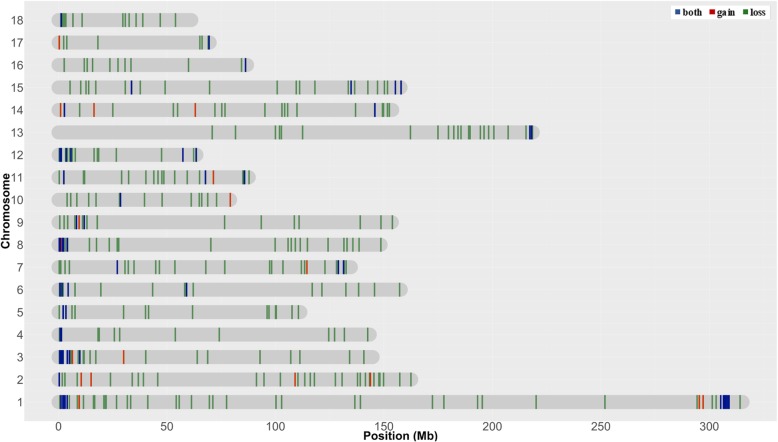
Chromosomal locations of 425 CNVRs identified along the 18 porcine autosomes

Although CNVRs were identified in all autosomes, the number and proportion of chromosomes covered by CNVRs varied considerably (Fig. 2, Table 1). Chromosome 1 showed the largest number of CNVRs (49 CNVRs), which covered only 3.94% of its sequence; the lowest coverage observed for a single chromosome. Although the SSC11 showed the highest coverage of a chromosome sequence (16.03%), this chromosome showed a small number of CNVR (22), but with bigger sizes. The SSC17 presented the smallest number of CNVR (10), covering only 5.05% of its sequence.

### Weighted single-step genome-wide association study

The estimated heritability for number of piglets born alive was 0.11 ± 0.008. The additive genetic direct and permanent environmental variances were 0.78 and 0.57, respectively, whereas the residual variance was 5.68.

**Fig. 3:**
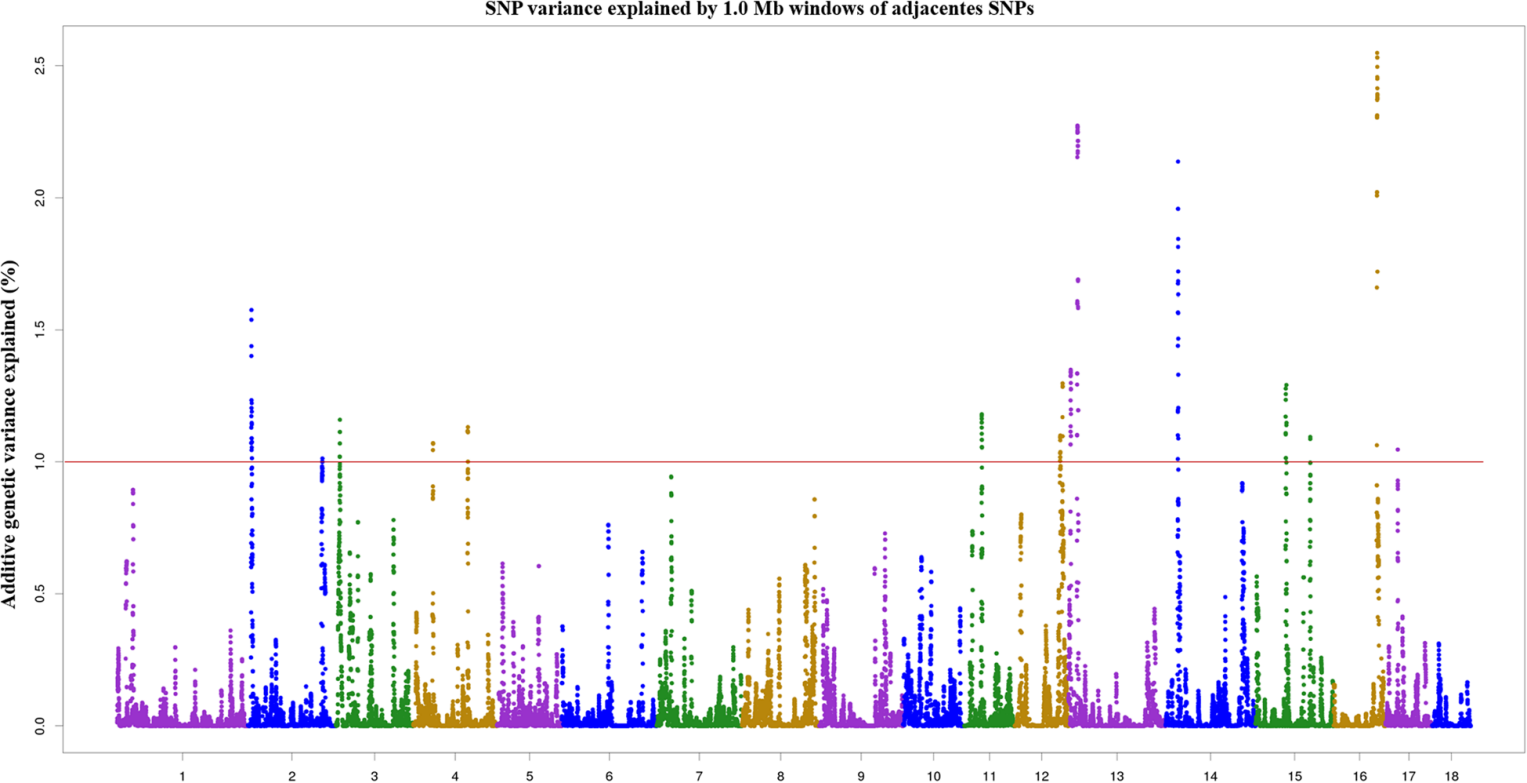
Proportion of additive genetic variance, explained by 1 Mb windows of adjacent SNPs, for number of piglets born alive

A total of 16 windows were detected each explaining more than 1% of the additive genetic variance for number of piglets born alive on chromosomes 2, 3, 4, 11, 12, 13, 14, 15, 16, and 17 (Fig. 3, Table 2). These significant windows explained a total of 22.54% of additive genetic variance for number of piglets born alive in the Duroc breed.

**Table 2:** Identification of protein-coding genes based on the additive genetic variance (Var) explained by 1 Mb windows of adjacent SNPs

Chr	Region (bp)	Genes	**Var**
SSC2^a^	4,239,252-5,222,613	*KDM2A, RHOD, C2H11orf86, SYT12, PC, RCE1, C2H11orf80, CCDC87, CCS, CTSF, ACTN3, ZDHHC24, BBS1, DPP3, PELI3, MRPL11, NPAS4, SLC29A2, B4GAT1, BRMS1, RIN1, CD248, TMEM151A, YIF1A, CNIH2, RAB1B, KLC2, PACS1, SF3B2*	1.57
SSC2	145,012,848-146,011,865	*SPOCK1, KLHL3, HNRNPA0, LOC100517462, MYOT, PKD2L2, LOC100520183, FAM13B, WNT8A, NME5, BRD8, KIF20A, CDC23, GFRA3, CDC25C, SLBP2*	1.01
SSC3^a^	3,981,003-4,980,011	*SDK1, FOXK1, AP5Z1, RADIL, PAPOLB, MMD2, WIPI2, SLC29A4, TNRC18, FBXL18, ACTB, FSCN1, RNF216*	1.16
SSC4	20,774,634-21,762,434	*COLEC10, TNFRSF11B, SAMD12, EXT1*	1.07
SSC4	104,642,633-105,640,667	*SLC39A1, CRTC2, DENND4B, GATAD2B, SLC27A3, INTS3, NPR1, ILF2, SNAPIN, CHTOP, S100A5, S100A4, S100A3, S100A2, S100A16, S100A14, S100A13, PPGRP-S, S100A9, S100A12, S100A8, LOC102161828, S100A7, PGLYRP3, LOR, PRR9, LELP1, SPRR2E, LOC100737840, SPRR1A, SPRP, IVL, SMCP, KPRP*	1.13
SSC11	25,254,733-26,232,874	*TNFSF11, AKAP11, DGKH, VWA8, RGCC, NAA16*	1.18
SSC12	54,066,400-55,065,792	*GP1BA, MINK1, PLD2, ZMYND15, CXCL16, MED11, ARRB2, PSMB6, GLTPD2, VMO1, TM4SF5, PELP1, ALOX15, SLC16A11, SLC16A13, BCL6B, C12H17orf49, RNASEK, ALOX12, CLEC10A, ASGR2, SLC2A4, CLDN7, ELP5, GABARAP, PHF23, DVL2, DLG4, YBX2, NEURL4, ACAP1, KCTD11, TMEM95, TNK1, PLSCR3, POLR2A, SLC35G6, ZBTB4, CHRNB1, FGF11, TMEM102, SPEM2, DNAH2*	1.10
SSC12^a^	56,619,955-57,608,007	*CCDC42, MFSD6L, PIK3R6, PIK3R5, NTN1, STX8, CFAP52, USP43, DHRS7C, GSG1L2, GLP2R, RCVRN, GAS7*	1.30
SSC13	3,171,064-4,162,557	*BTD, ANKRD28, GALNT15, DPH3, OXNAD1, RFTN1, DAZL*	1.35
SSC13	10,788,202-11,761,268	*UBE2E2*	2.27
SSC14	13,059,455-14,032,461	*SCARA5, NUGGC, ELP3, PNOC, ZNF395, FBXO16, FZD3, EXTL3, INTS9, HMBOX1, KIF13B*	2.14
SSC15	55,188,321-56,161,595	*FGFR1, LETM2, NSD3, PLPP5, DDHD2, BAG4, ADRB3, STAR, ASH2L, EIF4EBP1, LSM1, BRF2, ADGRA2, PLPBP, ERLIN2, ZNF703, KCNU1*	1.28
SSC15	57,551,770-58,532,159	*–*	1.29
SSC15	131,996,123-132,969,736	*TNS1, RUFY4, CXCR2, CXCR1*	1.09
SSC16	77,028,962-78,013,471	*GLRA1, G3BP1, ATOX1, LOC100514500, SPARC, FAT2, SLC36A1, SLC36A2, SLC36A3, GM2A, CCDC69, ANXA6*	2.55
SSC17^a^	17,325,007-18,300,615	*BMP2*	1.04

^a^region also identified in a CNVR

We identified common regions between GWAS and CNVR analyses on SSC2 (4.2–5.2 Mb), SSC3 (3.9–4.9 Mb), SSC12 (56.6–57.6 Mb), and SSC17 (17.3–18.3 Mb), comprising a total of 56 protein-coding genes (Table 2). These regions are very interesting because their gene content could affect the number of pigs born alive through changes in gene dosage. The windows on SSC2, SSC3 and SSC17 are in deletion CNVR, whereas the window on SSC12 is in deletion and duplication CNVR.

### Gene enrichment analysis

**Fig. 4:**
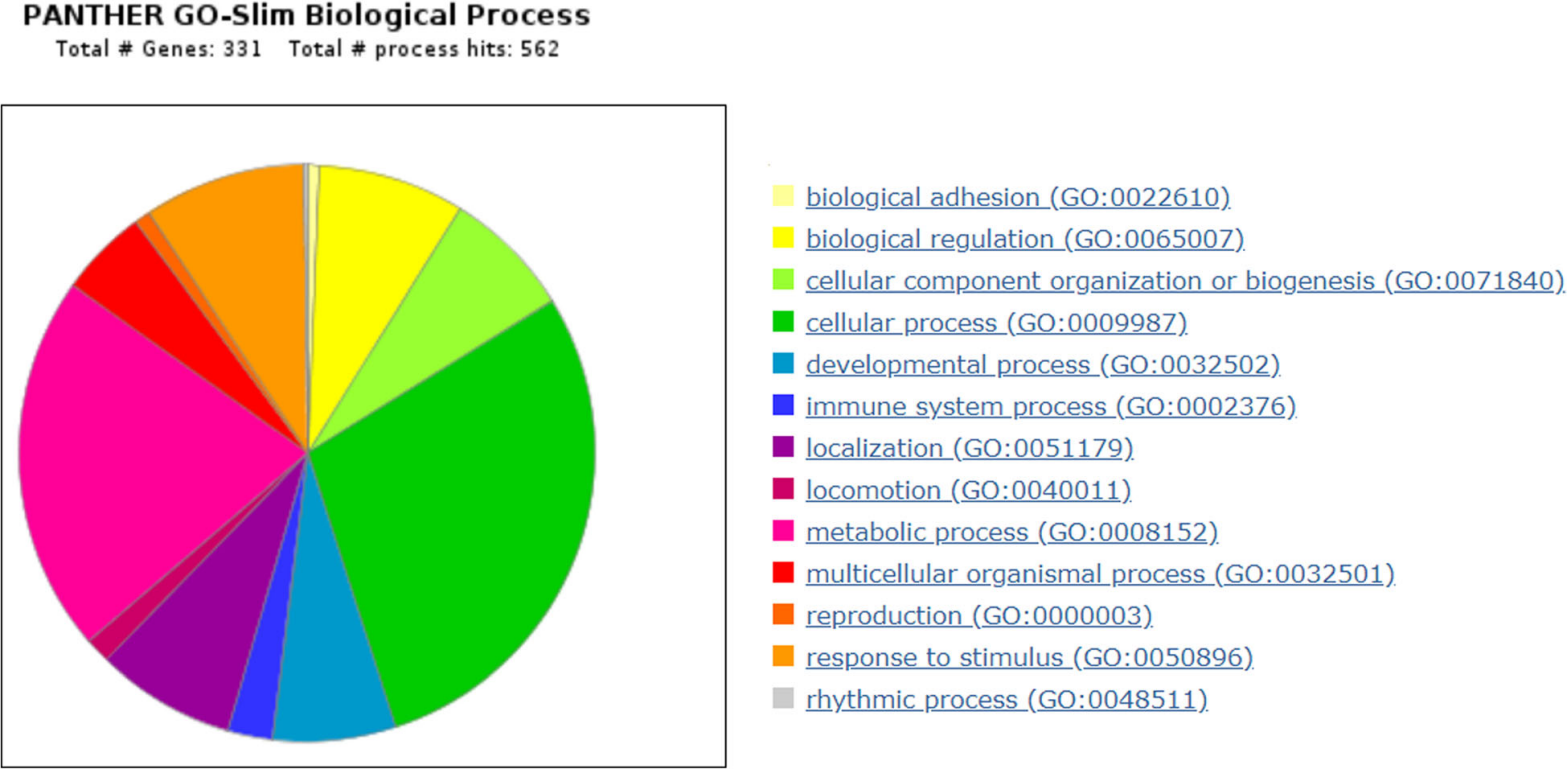
Pie chart representation of Panther Gene Ontology biological processes significantly over represented in this study. Genes were categorized into 13 biological processes, including biological adhesion (0.7%), biological regulation (8.2%), cellular component organization or biogenesis (7.3%), cellular process (28.8%), developmental process (6.9%), immune system process (2.5%), localization (7.8%), locomotion (1.4%), metabolic process (21.2%), multicellular organismal process (5.0%), reproduction (0.9%), response to stimulus (9.1%) and rhythmic process (0.2%)

Functional annotation revealed genes involved in 13 gene ontology (GO) biological processes such as reproduction (GO:0000003), developmental process (GO:0032502), cellular process (GO:0009987), cellular component organization or biogenesis (GO:0071840), biological regulation (GO:0065007), immune system process (GO:0002376), and rhythmic process (GO:0048511), which may play relevant roles in reproductive traits (Fig. 4, Additional file 2).

## Discussion

The number of animals used in this study is greater than previous in studies on pigs [12, 22, 23, 25, 27, 28, 37, 38], which could decrease the amount of false-positive CNVs.

Several studies also detected deletions more frequently than duplications in pigs [17, 18, 22, 23, 39] which could be partially explained by biological factors because non-allelic homologous recombination tends to create more deletions than duplications [40], and partially by technical bias because deletions tend to be under stronger purifying selection, i.e., deletions are more deleterious than duplications [41].

Although several studies have reported CNVs in the porcine genome, CNVs have been described to have breed-specific characteristics [22, 23, 25, 27, 28, 37, 39, 42]. By using the Illumina Porcine SNP60 BeadChip to detect CNVR, a total of 170 CNVRs (7 gains, 161 losses and 2 both) were detected in 293 Large White pigs [39], 249 CNVRs (70 gains, 43 losses and 136 both) in 585 Large White X Minzhu pigs [42], 65 CNVRs (21 gains, 32 losses and 12 both) in 223 Iberian pigs [37], and 348 CNVRs (88 gains, 243 losses and 17 both) in 302 animals from ten Chinese pig breeds [22]. By using arrayCGH technology, 37 CNVRs (18 gains and 19 losses) were identified in 12 Duroc boars [43]. Besides breed, it is important to highlight that the number of samples analyzed, as well as the differences in calling technology (NGS, SNP genotyping or arrayCGH), resolution, genome coverage and/or quality control to filter CNVs used in the aforementioned studies directly influence the results obtained, as described previously [44–46].

Some genes identified in common regions between GWAS and CNVR analyses were highlighted according to their function. The *KDM2A, ACTN3,* and *RHOD* genes are mapped on SSC2 (4.2–5.2 Mb) region. The *KDM2A* gene, also known as *FBXL11* and *JHDM1A*, plays an important role in gene silencing, cell cycle, and cell growth through histone demethylation modification. This gene was identified as differentially expressed in porcine embryonic skeletal muscle, being therefore involved in skeletal muscle development and growth [47]. The *ACTN3* gene also encodes a protein which exhibits an important function in muscle metabolism [48]. The *RHOD* gene is a regulator of reorganization of the actin cytoskeleton and consequently, regulates several cellular processes such as vesicle trafficking, chemotaxis, cell migration and proliferation [49].

The *ACTB* gene is located on SSC3 window (3.9–4.9 Mb). This gene encodes β-actin, a member of the actin family of proteins that are related to cell motility, structure, and integrity, fundamental processes for embryonic development. β-actin protein is required for meiosis in mouse oocytes [50] and for early embryonic development because knocking out this gene resulted in embryonic lethality [51].

According to its function, we identified some important genes mapped on SSC12 (56.6–57.6 Mb), such as *CCDC42*, *PIK3R5*, and *NTN1*. The *CCDC42* gene plays a crucial role in sperm development and male fertility in mouse [52], while the *PIK3R5* gene has been implicated in several cell functions such as proliferation, survival, differentiation, growth, motility, and intracellular trafficking [53, 54]. The *NTN1* gene codifies the netrin-1 which is an essential protein of embryonic development with important functions in cell migration, axon guidance, angiogenesis and morphogenesis [55]. Netrin-1 also is a crucial factor for regulating angiogenesis in placenta [56] and osteoclast differentiation [57]. Basini et al. [58] identified the *NTN1* gene as a potential modulator of swine follicular function.

The *BMP2* gene is the only gene mapped on SSC17 window (17.3–18.3 Mb). BMP proteins exhibit wide spectrum of activities in several tissues (cartilage, bone, blood vessels, liver, lung, kidney, heart and neurons) and perform multiple roles in regulation of growth, differentiation, and apoptosis, playing important functions during embryonic development and tissue morphogenesis [59]. The *BMP2* gene plays a critical role in mesenchymal cells influencing adipogenesis, myogenesis, chondrogenesis, and osteogenesis [60–63]. Homozygous knockout mice for *BMP2* gene exhibits embryonic lethality with defects in extra-embryonic and embryonic tissues [64], whereas heterozygous knockout mice have defects in cartilage, bone and heart development [63]. As the *BMP2* is mapped to a window that explains part of the additive genetic variance for number of piglets born alive, which is located on a deletion CNVR, the *BMP2* becomes an important gene to influence the number of piglets born alive, especially because subnormal levels of *BMP2* negatively impacted embryonic development [63] and in the adult, it is required for uterine decidual response during embryo implantation [65].

Additionally, we highlighted the SSC12 (54.0–55.0 Mb) and SSC14 (13.0–14.0 Mb) chromosome regions identified only by WssGWAS due its gene content. The *ZMYND15, YBX2* and *TMEM95* genes are mapped on SSC12 (54.0–55.0 Mb) and are related with reproductive traits. The *ZMYND15* gene is primordial for sperm production and male fertility [66]. The *YBX2* gene codifies a protein required for mammal development and fertility because knockout mice for this gene presented disruption of spermatogenesis and oogenesis [67–69]. The *TMEM95* gene codifies a protein located at the surface of spermatozoa and deficiency of *TMEM95* severely compromises male reproductive performance, resulting in subfertility in cattle [70]. The *ELP3* gene, mapped on SSC14 window (13.0–14.0 Mb), plays important roles in embryonic stem cell maintenance and early development in mouse [71].

The *SCIMP, TNP1, MUS81, KCNU1*, and *ILF2* genes were identified as related with reproduction (GO:0000003) process. Among these genes, we highlighted the *TNP1* gene which codifies a transition nuclear protein that acts in spermiogenesis. To detect the role of *TPN1* in vivo, a study produced knockout mice and identified that mutations had no effect on female fertility, although had reduced litter sizes [72]. The *KCNU1* gene, also known as *SLO3*, is expressed only in mammalian testis and plays an important role in male fertility [73].

Members of S100 protein family (*S100A3, S100A4, S100A5, S100A9, S100A12, S100A14, S100A16*) were identified acting in cellular process (GO:0009987) and metabolic process (GO:0008152). These genes act in the regulation of cellular processes such as differentiation, proliferation, motility, chemotaxis, apoptosis, and inflammatory response [74]. The *S100A14* and *S100A16* were identified as candidate genes for human embryo adhesion and implantation [75].

Deleted in Azoospermia (DAZ) gene family encodes proteins with essential roles in male and female gametogenesis. The protein encoded by *DAZL* gene, related with metabolic process (GO:0008152), was detected in fetal germ cells and developing oocytes [76]. Mutations in *DAZL* gene have been associated to infertility in man and women [77, 78].

The *FGF11, FGRF1* and *WNT8A* genes are related to developmental process (GO:0032502), response to stimulus (GO:0050896), and cellular process (GO:0009987). The *FGF11* and *FGRF1* gene encodes a member of the fibroblast growth factor (FGF) family and a member of the fibroblast growth factor receptor (FGFR) family, respectively. The FGF family members and their receptors influence mitogenesis and differentiation with essential roles in biological processes, including preimplantation of embryos, embryonic development, and organogenesis [79]. The *WNT8A* gene, expressed during early embryogenesis, belongs to WNT gene family that has been related to developmental processes, including differentiation, proliferation, apoptosis, regulation of cell fate and patterning during embryonic development [80].

The *TNK1, PNOC* and *AKAP11* genes are related with biological regulation (GO:0065007) and cellular process (GO:0009987). The *TNK1* gene encodes a tyrosine protein kinase family member highly expressed in fetal tissues signaling pathways widely utilized during fetal development [81]. Tyrosine protein kinases are crucial regulators of intracellular signal transduction pathways, cell growth, differentiation, survival, and migration [82]. The *AKAP11* gene is highly expressed during spermatogenesis and in mature sperm with assumed role in spermatogenesis and sperm functions, which may play functions in cell cycle control of germ and somatic cells [83, 84]. The *PNOC* gene encodes the precursor for biologically active peptides, such as nociceptin, which have been related to several physiological roles in the central nervous system. Additionally, a study suggested that nociceptin plays a key function in meiosis during spermatogenesis [85].

## Conclusion

In this study we integrated WssGWAS and CNV analyses to improve the investigation of genetic factors determining number born alive in Duroc pigs. Our study was the first to provide a map of 425 CNV regions in the pig genome, which is a substantial source of information for further studies that aim to explore the association between reproductive traits and CNV regions. The overlapping regions between WssGWAS and CNVR analyses harbor important causative variants related to pig reproductive traits based on their critical roles in fertilization, development of gametes and embryos, which may be valuable for additional validation and consideration in future selection programs aiming to improve number of piglets born alive and other reproductive traits.

## Methods

### Phenotype and pedigree information

The phenotypic information was collected by Smithfield Premium Genetics from five farms of Duroc pigs. Number of piglets born alive was recorded from sows born between 2008 and 2017. A total of 39,427 records from 13,845 sows spanning 1 to 12 parities were used, and number of piglets born alive in each parity ranged from 1 and 19. Animals were grouped into nine contemporary groups which were formed by concatenating farm, month and year of farrowing. Pedigree information was available for 772,779 animals.

### SNP genotyping and quality control

A total of 3892 DNA samples were genotyped using the GeneSeek® Genomic Profiler Porcine HD (https://support.illumina.com/downloads/geneseek-ggp-porcine-hd-product-files.html) which contained 68,528 SNPs across 18 autosomes and two sex chromosomes. Aiming to eliminate poor-quality DNA samples and decrease false-positive CNVs, only the samples with a call rate greater than 98% and call frequency greater than 90% were retained. The SNPs mapped to the sex chromosomes and those not mapped to any of the chromosomes were discarded. A total of 3520 samples genotyped for 57,962 SNPs remained after quality control for CNV and WssGWAS analyses.

### CNV detection and statistical analysis

Individual-based CNV detection was conducted using PennCNV software [86] based on a hidden Markov model, which is widely used for detecting CNV based on SNP array data due its relatively low false-positive rate [87].

Multiple sources of information were used simultaneously for obtaining accurate CNV detections such as distance between SNPs, log R ratio (LRR), population frequency of the B allele (PBF), and B allele frequency (BAF). The LRR and BAF measures were automatically computed by GenomeStudio software v2.0 (Illumina, Inc., USA) from the signal intensity files of the SNP data. The PBF file was calculated from the signal files using the compile_pbf.pl routine present in the PennCNV software [86]. In addition, we performed a wave adjustment procedure for genomic waves due to guanine-cytosine content effect applying the gcmodel option in the PennCNV software to eliminated false positive CNVs detected from the differentiating signal intensities generated by genomic waves [88, 89]. The porcine gcmodel was generated by calculating the guanine-cytosine content in genomic regions surrounding each SNP (500 kb each side).

A quality control of signal intensity was performed to reduce false-positive CNVs originated from poor-quality DNA and to increase the confidence in CNV identification, which included a BAF drift < 0.01, standard deviation of LRR < 0.30 and/or GC wave factor < 0.05 (after genomic waves were corrected by guanine-cytosine content) to generate raw CNV calls. The CNVs identified in only one sample and with less than three consecutive SNPs were discarded.

In order to eliminate inconsistent calling of CNV boundaries, CNV regions (CNVR) were inferred through concatenation of filtered individual CNVs identified in more than one animal. Regions with less than 0.5% allele frequency and with very low density of overlapping (recurrence < 0.1) were discarded for a more precise definition of CNVR.

### Weighted single-step genome-wide association study (WssGWAS)

Variance components for number of piglets born alive were estimated by AIREMLF90 software [90], which uses the Average Information Restricted Maximum Likelihood method to estimates variance components as well as solutions for fixed and random effects. The single-trait model included contemporary group as fixed effect, random animal genetic effect (containing inbreeding), permanent environmental effect, and the residual effect. In matrix notation, the model is described as:

$$ \mathbf{y}=\boldsymbol{Xb}+\boldsymbol{Wa}+\boldsymbol{Kpe}+\boldsymbol{e} $$

where **y** is the vector of phenotypic records; ***b*** is the vector of fixed effect of contemporary groups; ***a*** is the vector of direct additive genetic effects, ***pe*** is the vector of permanent environmental effects; and ***X***, **W**, and ***K*** are the incidence matrices for the effects contained in ***b***, ***a***, and ***pe***, respectively. Narrow sense heritability was estimated as ${\boldsymbol{h}}^{\mathbf{2}}=\frac{{\boldsymbol{\sigma}}_{\boldsymbol{a}}^{\mathbf{2}}}{{\boldsymbol{\sigma}}_{\boldsymbol{a}}^{\mathbf{2}}+{\boldsymbol{\sigma}}_{\boldsymbol{pe}}^{\mathbf{2}}+{\boldsymbol{\sigma}}_{\boldsymbol{e}}^{\mathbf{2}}}$, where ${\sigma}_i^2$ is the variance of the *i*-th effect.

The same animal model as described previously was used to estimate the genomic breeding values using the ssGBLUP (single-step genomic BLUP) approach [91], which combines genomic and pedigree relationships into a realized relationship matrix (**H**). Therefore, the difference between the regular BLUP and ssGBLUP is that the inverse of the pedigree relationship matrix (**A**^− 1^) is replaced by **H**^− 1^, which is represented as follows:

$$ {\mathbf{H}}^{-\mathbf{1}}={\mathbf{A}}^{-1}+\left[\begin{array}{cc}0& 0\\ {}0& {\mathbf{G}}^{-1}-{{\mathbf{A}}_{22}}^{-1}\end{array}\right] $$

where **G**^−1^ is the inverse of the genomic relationship matrix and **A**_22_^−1^ is the inverse pedigree relationship matrix for genotyped animals. The **G** matrix was constructed as in [30]:

$$ \mathbf{G}=\frac{{\mathbf{ZDZ}}^{\hbox{'}}}{2\sum {\mathrm{p}}_{\mathrm{i}}\left(1-{\mathrm{p}}_{\mathrm{i}}\right)}, $$

where **Z** is a matrix of genotypes centered by twice the current allele frequencies of each SNP (*p*); *i* is the *i*th locus; **D** is a diagonal matrix of weights (variances) for SNP, which is an identity matrix for the regular ssGBLUP. To avoid singularity problems, **G** was blended with 5% of **A**_22_.

After genomic breeding values (GEBV) were estimated by ssGBLUP, they were back solved to obtain SNP effects as described by [33]:

$$ \widehat{\boldsymbol{u}}=\boldsymbol{\uplambda} \mathbf{D}{\mathbf{Z}}^{\prime }{\mathbf{G}}^{-\mathbf{1}}{\widehat{\boldsymbol{a}}}_{\boldsymbol{g}} $$

where ${\widehat{\boldsymbol{a}}}_{\boldsymbol{g}}$ is GEBV for genotyped animals; ***λ*** is the ratio of SNP to additive genetic variances ($\frac{{\boldsymbol{\sigma}}_{\boldsymbol{u}}^{\mathbf{2}}}{{\boldsymbol{\sigma}}_{\boldsymbol{a}}^{\mathbf{2}}}=\frac{\mathbf{1}}{\sum_{\boldsymbol{i}=\mathbf{1}}^{\boldsymbol{M}}\mathbf{2}{\boldsymbol{p}}_{\boldsymbol{i}}\ \left(\mathbf{1}-{\boldsymbol{p}}_{\boldsymbol{i}}\right)\ }$). The weight for each SNP was calculated based on SNP effects as follows [33]:

$$ {\boldsymbol{d}}_{\boldsymbol{i}}={\widehat{\boldsymbol{u}}}_{\boldsymbol{i}}^{\mathbf{2}}\ \mathbf{2}{\boldsymbol{p}}_{\boldsymbol{i}}\ \left(\mathbf{1}-{\boldsymbol{p}}_{\boldsymbol{i}}\right) $$

where ***d***_***i***_ is the weight for the *i*-th SNP.

The WssGWAS is an iterative procedure that involves the following steps [33]: (i) set **D** = **I**; (ii) construct **G** matrix as described by [30]; (iii) estimate GEBVs for all animals using ssGBLUP; (iv) estimate the SNP effect; (v) estimate weight for each SNP individually; (vi) normalize **D** to maintain the additive genetic variance constant; (vii) iterate from step ii.

The analyses were performed using BLUPF90 software [92] and the results were obtained as the percentage of additive genetic variance explained by 1 Mb sliding SNP-windows. The percentage of the additive genetic variance explained by ***i***^th^ window was calculated as in [34]:

$$ \frac{\boldsymbol{\operatorname{var}}\left({\boldsymbol{a}}_{\boldsymbol{i}}\right)}{{\boldsymbol{\sigma}}_{\boldsymbol{a}}^{\mathbf{2}}}\ast \mathbf{1}\mathbf{00}=\frac{\boldsymbol{\operatorname{var}}\ \left({\sum}_{\boldsymbol{j}=\mathbf{1}}^{\boldsymbol{n}}{\boldsymbol{z}}_{\boldsymbol{j}}{\widehat{\boldsymbol{u}}}_{\boldsymbol{j}}\right)\ }{{\boldsymbol{\sigma}}_{\boldsymbol{a}}^{\mathbf{2}}}\ast \mathbf{1}\mathbf{00} $$

where ***a***_***i***_ is genetic value of the ***i***-th region consisting of 1 Mb window length physical size, ${\boldsymbol{\sigma}}_{\boldsymbol{a}}^{\mathbf{2}}$ is the total genetic variance, ***z***_***j***_ is vector of genotype of the ***j***-th SNP for all animals, ${\widehat{\boldsymbol{u}}}_{\boldsymbol{j}}$ is SNP effect of the ***j***-th SNP within the ***i***-th region, ***n*** is the number of SNP in a window of 1 Mb.

### Gene annotation and enrichment analysis

The genomic regions exhibiting more than 1% of the additive genetic variance were prospected for possible QTL related to number of piglets born alive. The gene content of the windows was identified using the Ensembl Biomart tool [93]. The search for biologically relevant functions was performed with Panther v.13.1 database [94] selecting a 500 Kb window around a significant region (upstream and downstream) and using Sscrofa10.2 assembly as genome reference. *P*-values generated by Panther were Bonferonni-corrected for the number of conducted comparisons.

## Additional files

Additional file 1: Description of 425 CNVRs detected in the porcine genome. (DOCX 75 kb)

Additional file 2: Gene ontology biological processes revealed by Panther analysis. (DOCX 40 kb)

## Abbreviations

BAF: Ballele frequency
BLUP: Best linear unbiased prediction
CNV: Copy number variation
CNVR: Copy number variation region
GO: Gene ontology
GWAS: Genome-wide association study
LRR: Log R ratio
NGS: Next generation sequencing
PBF: Population frequency of the B allele
QTL: Quantitative trait loci
SNP: Single nucleotide polymorphism
SSC: *Sus scrofa* chromosome
ssGBLUP: Single-step genomic best linear unbiased prediction
ssGWAS: Single-step genomic association study
WssGWAS: Weighted single-step approach for genome-wide association study
