# Supplementary material for: A genome-wide single nucleotide polymorphism and copy number variation analysis for number of piglets born alive
Source: BMC Genomics. 2019 Apr 27;20:321. doi: 10.1186/s12864-019-5687-0 (PMC6487013; doi:10.1186/s12864-019-5687-0)
Supplement: Supplementary file 1 — Description of 425 CNVRs detected in the porcine genome. (DOCX 75 kb) [file 12864_2019_5687_MOESM1_ESM.docx]

**Additional file 1:** Description of 425 CNVRs detected in the porcine genome

| **SSC** | **Start (bp)** | **End (bp)** | **Length (bp)** | **Status** | **Number of samples** |
| --- | --- | --- | --- | --- | --- |
| 1 | 233515 | 778942 | 545427 | loss | 428 |
| 1 | 778943 | 791056 | 12113 | both | 430 |
| 1 | 791057 | 1637441 | 846384 | loss | 428 |
| 1 | 1637442 | 1843489 | 206047 | both | 437 |
| 1 | 2010349 | 2073619 | 63270 | both | 430 |
| 1 | 2667200 | 2812415 | 145215 | both | 508 |
| 1 | 3640815 | 3691600 | 50785 | both | 430 |
| 1 | 4630315 | 4690022 | 59707 | loss | 584 |
| 1 | 8342840 | 8410340 | 67500 | loss | 20 |
| 1 | 9189551 | 9221985 | 32434 | gain | 49 |
| 1 | 11153176 | 11270636 | 117460 | loss | 21 |
| 1 | 15775179 | 15812314 | 37135 | loss | 37 |
| 1 | 16313130 | 16423967 | 110837 | loss | 69 |
| 1 | 20559479 | 20646252 | 86773 | loss | 147 |
| 1 | 21295161 | 21473869 | 178708 | loss | 14 |
| 1 | 21576278 | 21877265 | 300987 | loss | 143 |
| 1 | 26383627 | 26856773 | 473146 | loss | 248 |
| 1 | 31363404 | 31644375 | 280971 | loss | 28 |
| 1 | 32902685 | 33019987 | 117302 | loss | 17 |
| 1 | 40795695 | 41172639 | 376944 | loss | 19 |
| 1 | 53913932 | 54144185 | 230253 | loss | 18 |
| 1 | 55290662 | 55354812 | 64150 | loss | 27 |
| 1 | 60938935 | 61159644 | 220709 | loss | 16 |
| 1 | 69193400 | 70068760 | 875360 | loss | 21 |
| 1 | 70784564 | 70920102 | 135538 | loss | 140 |
| 1 | 77121687 | 78587646 | 1465959 | loss | 848 |
| 1 | 99897622 | 100420304 | 522682 | loss | 14 |
| 1 | 102484726 | 102718610 | 233884 | loss | 14 |
| 1 | 136240599 | 136502346 | 261747 | loss | 17 |
| 1 | 138810037 | 138927010 | 116973 | loss | 61 |
| 1 | 172013706 | 172291082 | 277376 | loss | 83 |
| 1 | 177284527 | 177412813 | 128286 | loss | 22 |
| 1 | 192820604 | 192978414 | 157810 | loss | 1836 |
| 1 | 194987488 | 195271689 | 284201 | loss | 69 |
| 1 | 219816097 | 220815008 | 998911 | loss | 20 |
| 1 | 251512794 | 251788183 | 275389 | loss | 29 |
| 1 | 294012711 | 294129324 | 116613 | loss | 21 |
| 1 | 295017152 | 295109980 | 92828 | gain | 18 |
| 1 | 296787813 | 296820153 | 32340 | gain | 27 |
| 1 | 300989453 | 301183660 | 194207 | loss | 16 |
| 1 | 302771100 | 303425035 | 653935 | loss | 122 |
| 1 | 304891807 | 304958591 | 66784 | both | 442 |
| 1 | 306141661 | 306198036 | 56375 | both | 442 |
| 1 | 306704173 | 306721092 | 16919 | both | 487 |
| 1 | 307000519 | 307109845 | 109326 | both | 441 |
| 1 | 307419517 | 307718072 | 298555 | both | 580 |
| 1 | 308140434 | 308170662 | 30228 | both | 455 |
| 1 | 308716258 | 308783518 | 67260 | both | 442 |
| 1 | 313808410 | 314134964 | 326554 | loss | 121 |
| 2 | 0 | 16416 | 16416 | both | 191 |
| 2 | 16417 | 632707 | 616290 | loss | 182 |
| 2 | 1385905 | 1650841 | 264936 | loss | 33 |
| 2 | 2599113 | 7209835 | 4610722 | loss | 156 |
| 2 | 8270193 | 8674597 | 404404 | loss | 17 |
| 2 | 10115081 | 10209820 | 94739 | gain | 31 |
| 2 | 14662231 | 14722820 | 60589 | gain | 46 |
| 2 | 23588944 | 25881871 | 2292927 | loss | 166 |
| 2 | 33623071 | 33784719 | 161648 | loss | 48 |
| 2 | 36428198 | 36702202 | 274004 | loss | 25 |
| 2 | 38779149 | 39302035 | 522886 | loss | 60 |
| 2 | 45377267 | 45986058 | 608791 | loss | 91 |
| 2 | 90892618 | 91076410 | 183792 | loss | 43 |
| 2 | 94358769 | 94631894 | 273125 | loss | 160 |
| 2 | 101918372 | 103098050 | 1179678 | loss | 42 |
| 2 | 108692886 | 108726361 | 33475 | gain | 32 |
| 2 | 110005387 | 110423447 | 418060 | loss | 467 |
| 2 | 113160574 | 113226899 | 66325 | loss | 218 |
| 2 | 115691979 | 116470460 | 778481 | loss | 555 |
| 2 | 117593190 | 117929110 | 335920 | loss | 21 |
| 2 | 127266681 | 127347749 | 81068 | loss | 17 |
| 2 | 130371168 | 130487175 | 116007 | loss | 84 |
| 2 | 137484909 | 137652218 | 167309 | loss | 54 |
| 2 | 138737642 | 139418070 | 680428 | loss | 156 |
| 2 | 141118802 | 141272522 | 153720 | loss | 54 |
| 2 | 143019258 | 143262924 | 243666 | loss | 22 |
| 2 | 143423036 | 143480924 | 57888 | gain | 79 |
| 2 | 145036045 | 145159677 | 123632 | loss | 22 |
| 2 | 147317430 | 147456334 | 138904 | loss | 99 |
| 2 | 147771918 | 148001179 | 229261 | loss | 43 |
| 2 | 149483880 | 149861716 | 377836 | loss | 46 |
| 2 | 156951510 | 158488428 | 1536918 | loss | 146 |
| 2 | 162084552 | 162298086 | 213534 | loss | 71 |
| 3 | 56943 | 240240 | 183297 | loss | 1034 |
| 3 | 240241 | 364888 | 124647 | both | 1061 |
| 3 | 364889 | 667215 | 302326 | loss | 1034 |
| 3 | 667216 | 1045800 | 378584 | both | 1105 |
| 3 | 1045801 | 1243957 | 198156 | loss | 1034 |
| 3 | 1243958 | 1368601 | 124643 | both | 1113 |
| 3 | 1368602 | 1695804 | 327202 | loss | 1034 |
| 3 | 1695805 | 1797693 | 101888 | both | 1044 |
| 3 | 1797694 | 3709764 | 1912070 | loss | 1034 |
| 3 | 3709765 | 3760847 | 51082 | both | 1046 |
| 3 | 3760848 | 4692552 | 931704 | loss | 1034 |
| 3 | 4692553 | 4733469 | 40916 | both | 1040 |
| 3 | 4733470 | 4748899 | 15429 | loss | 1034 |
| 3 | 5435898 | 5570582 | 134684 | loss | 27 |
| 3 | 6048594 | 6096139 | 47545 | gain | 22 |
| 3 | 8728505 | 9463667 | 735162 | loss | 25 |
| 3 | 9463668 | 9491312 | 27644 | both | 34 |
| 3 | 11090073 | 11332375 | 242302 | loss | 17 |
| 3 | 11428687 | 11519897 | 91210 | loss | 37 |
| 3 | 14198476 | 14341973 | 143497 | loss | 14 |
| 3 | 16839756 | 17428593 | 588837 | loss | 18 |
| 3 | 29676368 | 29729288 | 52920 | gain | 17 |
| 3 | 39858459 | 40047077 | 188618 | loss | 21 |
| 3 | 63608453 | 64022095 | 413642 | loss | 44 |
| 3 | 68404896 | 70024504 | 1619608 | loss | 492 |
| 3 | 92529004 | 92750092 | 221088 | loss | 243 |
| 3 | 106694292 | 107151059 | 456767 | loss | 41 |
| 3 | 110903404 | 111512674 | 609270 | loss | 20 |
| 3 | 133824893 | 134191627 | 366734 | loss | 16 |
| 3 | 140401208 | 142396701 | 1995493 | loss | 166 |
| 4 | 95368 | 425397 | 330029 | loss | 680 |
| 4 | 425398 | 456762 | 31364 | both | 687 |
| 4 | 456763 | 969778 | 513015 | loss | 680 |
| 4 | 969779 | 1037907 | 68128 | both | 700 |
| 4 | 1037908 | 3046732 | 2008824 | loss | 680 |
| 4 | 17865227 | 18008464 | 143237 | loss | 36 |
| 4 | 18393679 | 18502471 | 108792 | loss | 18 |
| 4 | 25384415 | 27265073 | 1880658 | loss | 143 |
| 4 | 27832880 | 29731872 | 1898992 | loss | 144 |
| 4 | 53485416 | 54438696 | 953280 | loss | 248 |
| 4 | 73747755 | 74095578 | 347823 | loss | 19 |
| 4 | 124205654 | 124473177 | 267523 | loss | 72 |
| 4 | 126897791 | 128153004 | 1255213 | loss | 395 |
| 4 | 131423303 | 131918945 | 495642 | loss | 113 |
| 4 | 142168382 | 142431371 | 262989 | loss | 30 |
| 5 | 2847 | 1787761 | 1784914 | loss | 388 |
| 5 | 1787762 | 1853896 | 66134 | both | 391 |
| 5 | 1853897 | 3057476 | 1203579 | loss | 388 |
| 5 | 3057477 | 3095194 | 37717 | both | 393 |
| 5 | 3095195 | 3136770 | 41575 | loss | 388 |
| 5 | 5857867 | 6387176 | 529309 | loss | 99 |
| 5 | 7253925 | 8052208 | 798283 | loss | 19 |
| 5 | 29640202 | 29952075 | 311873 | loss | 17 |
| 5 | 39749166 | 40704565 | 955399 | loss | 192 |
| 5 | 41151243 | 41617274 | 466031 | loss | 35 |
| 5 | 61439142 | 61561329 | 122187 | loss | 27 |
| 5 | 95787046 | 96118566 | 331520 | loss | 240 |
| 5 | 96667656 | 96985476 | 317820 | loss | 21 |
| 5 | 99696730 | 99972762 | 276032 | loss | 14 |
| 5 | 100012561 | 100755400 | 742839 | loss | 22 |
| 5 | 107283071 | 107723771 | 440700 | loss | 169 |
| 5 | 110220891 | 111027924 | 807033 | loss | 208 |
| 6 | 127259 | 339910 | 212651 | loss | 466 |
| 6 | 339911 | 504970 | 165059 | both | 814 |
| 6 | 504971 | 1122428 | 617457 | loss | 466 |
| 6 | 1122428 | 1281225 | 158797 | loss | 27 |
| 6 | 1281226 | 1593899 | 312673 | both | 93 |
| 6 | 1775500 | 2899443 | 1123943 | loss | 286 |
| 6 | 2899444 | 2952600 | 53156 | BOTH | 316 |
| 6 | 2952601 | 4085043 | 1132442 | LOSS | 286 |
| 6 | 4085044 | 4204135 | 119091 | both | 405 |
| 6 | 7245900 | 7399896 | 153996 | loss | 26 |
| 6 | 19157647 | 19350290 | 192643 | loss | 63 |
| 6 | 43031042 | 43276891 | 245849 | loss | 30 |
| 6 | 57809637 | 58614351 | 804714 | loss | 160 |
| 6 | 58614352 | 58753925 | 139573 | both | 269 |
| 6 | 58753926 | 59962739 | 1208813 | loss | 160 |
| 6 | 61748636 | 62399306 | 650670 | loss | 391 |
| 6 | 116602688 | 118862387 | 2259699 | loss | 22 |
| 6 | 121067169 | 122244581 | 1177412 | loss | 583 |
| 6 | 132116850 | 133090986 | 974136 | loss | 1533 |
| 6 | 137985648 | 138170945 | 185297 | loss | 570 |
| 6 | 145316210 | 145382280 | 66070 | loss | 14 |
| 6 | 156873906 | 157413487 | 539581 | loss | 55 |
| 7 | 0 | 208384 | 208384 | loss | 36 |
| 7 | 684039 | 2431990 | 1747951 | loss | 201 |
| 7 | 2659265 | 3596224 | 936959 | loss | 82 |
| 7 | 4658272 | 4697201 | 38929 | loss | 33 |
| 7 | 26738482 | 26822610 | 84128 | both | 22 |
| 7 | 30277462 | 30340595 | 63133 | loss | 19 |
| 7 | 31800221 | 32499186 | 698965 | loss | 25 |
| 7 | 34450700 | 34925816 | 475116 | loss | 180 |
| 7 | 44467498 | 45185966 | 718468 | loss | 44 |
| 7 | 46149418 | 46203060 | 53642 | loss | 19 |
| 7 | 53302953 | 53455645 | 152692 | loss | 59 |
| 7 | 67550289 | 67797696 | 247407 | loss | 338 |
| 7 | 76291007 | 76412419 | 121412 | loss | 837 |
| 7 | 96970994 | 97136628 | 165634 | loss | 57 |
| 7 | 97889360 | 98033733 | 144373 | loss | 77 |
| 7 | 103101452 | 103232787 | 131335 | loss | 63 |
| 7 | 111591929 | 111831499 | 239570 | loss | 67 |
| 7 | 113081934 | 113391764 | 309830 | loss | 18 |
| 7 | 114142861 | 114240807 | 97946 | gain | 15 |
| 7 | 122473934 | 122644975 | 171041 | loss | 21 |
| 7 | 127789154 | 128614045 | 824891 | loss | 474 |
| 7 | 128614046 | 128692840 | 78794 | both | 487 |
| 7 | 128692841 | 131034869 | 2342028 | loss | 474 |
| 7 | 131034870 | 131336733 | 301863 | both | 840 |
| 7 | 131336734 | 131388532 | 51798 | loss | 474 |
| 7 | 132215659 | 132468484 | 252825 | loss | 56 |
| 8 | 4941 | 11533 | 6592 | loss | 410 |
| 8 | 11534 | 36119 | 24585 | both | 414 |
| 8 | 36120 | 267312 | 231192 | loss | 410 |
| 8 | 267313 | 283598 | 16285 | both | 423 |
| 8 | 283599 | 514314 | 230715 | loss | 410 |
| 8 | 514315 | 580533 | 66218 | both | 424 |
| 8 | 580534 | 970500 | 389966 | loss | 410 |
| 8 | 970501 | 1021698 | 51198 | gain | 39 |
| 8 | 1358643 | 1569525 | 210882 | loss | 83 |
| 8 | 1569526 | 1653880 | 84354 | both | 100 |
| 8 | 1653881 | 1761011 | 107130 | loss | 83 |
| 8 | 1761012 | 1884635 | 123623 | both | 85 |
| 8 | 1884636 | 2041191 | 156555 | loss | 83 |
| 8 | 2041192 | 2084589 | 43397 | both | 86 |
| 8 | 2084590 | 2104157 | 19567 | loss | 83 |
| 8 | 3016722 | 3769688 | 752966 | loss | 209 |
| 8 | 3769689 | 3807765 | 38076 | both | 222 |
| 8 | 3807766 | 4744793 | 937027 | loss | 209 |
| 8 | 13891731 | 14014229 | 122498 | loss | 23 |
| 8 | 17154765 | 17295697 | 140932 | loss | 29 |
| 8 | 23033602 | 24033377 | 999775 | loss | 163 |
| 8 | 26713659 | 27215523 | 501864 | loss | 73 |
| 8 | 27396362 | 27724935 | 328573 | loss | 359 |
| 8 | 69865381 | 70033358 | 167977 | loss | 44 |
| 8 | 99493331 | 99661929 | 168598 | loss | 96 |
| 8 | 105457647 | 106292634 | 834987 | loss | 68 |
| 8 | 106921098 | 107185764 | 264666 | loss | 142 |
| 8 | 108799389 | 108903615 | 104226 | loss | 29 |
| 8 | 111078280 | 111286492 | 208212 | loss | 55 |
| 8 | 114376195 | 114407527 | 31332 | loss | 278 |
| 8 | 123895414 | 123996880 | 101466 | loss | 60 |
| 8 | 131207688 | 131694842 | 487154 | loss | 135 |
| 8 | 132645572 | 132890624 | 245052 | loss | 18 |
| 8 | 135332909 | 137509645 | 2176736 | loss | 126 |
| 8 | 138190631 | 138408069 | 217438 | loss | 113 |
| 8 | 148242825 | 148440405 | 197580 | loss | 226 |
| 9 | 232541 | 1562921 | 1330380 | loss | 144 |
| 9 | 2241284 | 2815486 | 574202 | loss | 19 |
| 9 | 3870433 | 4086982 | 216549 | loss | 81 |
| 9 | 7178792 | 7926116 | 747324 | loss | 41 |
| 9 | 7926117 | 7928669 | 2552 | both | 43 |
| 9 | 7928670 | 8122171 | 193501 | loss | 41 |
| 9 | 9151597 | 9224376 | 72779 | gain | 96 |
| 9 | 10701263 | 11488055 | 786792 | loss | 52 |
| 9 | 11488056 | 11547115 | 59059 | both | 54 |
| 9 | 11547116 | 12418244 | 871128 | loss | 52 |
| 9 | 12756823 | 12830635 | 73812 | loss | 29 |
| 9 | 17512724 | 18631113 | 1118389 | loss | 49 |
| 9 | 76198310 | 76692830 | 494520 | loss | 95 |
| 9 | 93098631 | 96564969 | 3466338 | loss | 43 |
| 9 | 108348177 | 108576795 | 228618 | loss | 104 |
| 9 | 110530933 | 111817735 | 1286802 | loss | 548 |
| 9 | 138738690 | 139299087 | 560397 | loss | 30 |
| 9 | 148280848 | 148877122 | 596274 | loss | 29 |
| 9 | 153542132 | 153656647 | 114515 | loss | 22 |
| 10 | 3605908 | 4040570 | 434662 | loss | 315 |
| 10 | 5282479 | 5608014 | 325535 | loss | 93 |
| 10 | 8094461 | 8176746 | 82285 | loss | 19 |
| 10 | 13549418 | 13589557 | 40139 | loss | 33 |
| 10 | 16942280 | 17082514 | 140234 | loss | 16 |
| 10 | 27756285 | 28202357 | 446072 | loss | 117 |
| 10 | 28202358 | 28248056 | 45698 | both | 159 |
| 10 | 28248057 | 29237613 | 989556 | loss | 117 |
| 10 | 39272858 | 39507554 | 234696 | loss | 122 |
| 10 | 47410091 | 47524154 | 114063 | loss | 63 |
| 10 | 60832812 | 61503252 | 670440 | loss | 24 |
| 10 | 64535089 | 64566072 | 30983 | loss | 46 |
| 10 | 65668997 | 66898636 | 1229639 | loss | 17 |
| 10 | 68422702 | 68582444 | 159742 | loss | 28 |
| 10 | 72501340 | 75964121 | 3462781 | loss | 143 |
| 10 | 78825911 | 78934427 | 108516 | gain | 14 |
| 11 | 244 | 2090930 | 2090686 | loss | 144 |
| 11 | 2090931 | 2128063 | 37132 | both | 146 |
| 11 | 2128064 | 2151176 | 23112 | loss | 144 |
| 11 | 11150563 | 11250799 | 100236 | loss | 70 |
| 11 | 11663909 | 11891740 | 227831 | loss | 106 |
| 11 | 28740570 | 29212417 | 471847 | loss | 128 |
| 11 | 31956957 | 32154968 | 198011 | loss | 62 |
| 11 | 39919176 | 42118356 | 2199180 | loss | 27 |
| 11 | 43582814 | 44061466 | 478652 | loss | 38 |
| 11 | 45502374 | 45992991 | 490617 | loss | 91 |
| 11 | 47282526 | 47452970 | 170444 | loss | 14 |
| 11 | 48200175 | 48299203 | 99028 | loss | 825 |
| 11 | 53204914 | 53388276 | 183362 | loss | 34 |
| 11 | 58988824 | 62480762 | 3491938 | loss | 56 |
| 11 | 64674579 | 65203181 | 528602 | loss | 37 |
| 11 | 67375641 | 67422576 | 46935 | both | 81 |
| 11 | 67422577 | 67548214 | 125637 | loss | 73 |
| 11 | 71047777 | 71641281 | 593504 | gain | 263 |
| 11 | 84599400 | 85287749 | 688349 | loss | 198 |
| 11 | 85287750 | 85498495 | 210745 | both | 226 |
| 11 | 85498496 | 86896318 | 1397822 | loss | 198 |
| 11 | 87470404 | 87674976 | 204572 | loss | 14 |
| 12 | 0 | 320417 | 320417 | loss | 635 |
| 12 | 320418 | 437614 | 117196 | both | 642 |
| 12 | 437615 | 490193 | 52578 | loss | 635 |
| 12 | 490194 | 634357 | 144163 | both | 721 |
| 12 | 634358 | 884329 | 249971 | loss | 635 |
| 12 | 884330 | 985539 | 101209 | both | 648 |
| 12 | 985540 | 2445965 | 1460425 | loss | 635 |
| 12 | 2922849 | 3211881 | 289032 | loss | 126 |
| 12 | 3211882 | 3290782 | 78900 | both | 166 |
| 12 | 3290783 | 3522238 | 231455 | loss | 126 |
| 12 | 3739024 | 4200376 | 461352 | loss | 131 |
| 12 | 4801182 | 5366301 | 565119 | loss | 27 |
| 12 | 5366302 | 5491557 | 125255 | both | 31 |
| 12 | 5491558 | 5645079 | 153521 | loss | 27 |
| 12 | 6028598 | 6484229 | 455631 | loss | 47 |
| 12 | 7385966 | 7800339 | 414373 | loss | 16 |
| 12 | 16041029 | 17081939 | 1040910 | loss | 20 |
| 12 | 17561137 | 17667874 | 106737 | loss | 89 |
| 12 | 18147178 | 18770184 | 623006 | loss | 103 |
| 12 | 26278595 | 26371789 | 93194 | loss | 15 |
| 12 | 47149478 | 47396819 | 247341 | loss | 29 |
| 12 | 56986300 | 57051011 | 64711 | both | 18 |
| 12 | 61949270 | 63095061 | 1145791 | loss | 108 |
| 12 | 63095062 | 63211419 | 116357 | both | 130 |
| 12 | 63211420 | 63239985 | 28565 | loss | 108 |
| 13 | 70499908 | 70665134 | 165226 | loss | 24 |
| 13 | 81236265 | 81437311 | 201046 | loss | 67 |
| 13 | 99619213 | 100035462 | 416249 | loss | 118 |
| 13 | 101512473 | 101786814 | 274341 | loss | 18 |
| 13 | 102377734 | 102458997 | 81263 | loss | 477 |
| 13 | 112158346 | 112770809 | 612463 | loss | 36 |
| 13 | 161870403 | 161962013 | 91610 | loss | 146 |
| 13 | 174559099 | 174758344 | 199245 | loss | 15 |
| 13 | 179392747 | 179550355 | 157608 | loss | 306 |
| 13 | 181846347 | 182461012 | 614665 | loss | 19 |
| 13 | 183789224 | 183883486 | 94262 | loss | 25 |
| 13 | 185168719 | 186455702 | 1286983 | loss | 150 |
| 13 | 188700141 | 188906506 | 206365 | loss | 416 |
| 13 | 189348927 | 189695982 | 347055 | loss | 174 |
| 13 | 194099336 | 194470218 | 370882 | loss | 121 |
| 13 | 195800128 | 197449273 | 1649145 | loss | 186 |
| 13 | 197909095 | 198408040 | 498945 | loss | 26 |
| 13 | 200335858 | 201166260 | 830402 | loss | 337 |
| 13 | 206903047 | 207119934 | 216887 | loss | 16 |
| 13 | 215147277 | 216907305 | 1760028 | loss | 580 |
| 13 | 216907306 | 217122230 | 214924 | both | 661 |
| 13 | 217122231 | 217743120 | 620889 | loss | 580 |
| 13 | 217743121 | 218111693 | 368572 | both | 812 |
| 13 | 218111694 | 218612146 | 500452 | loss | 580 |
| 14 | 610552 | 668579 | 58027 | gain | 21 |
| 14 | 2301796 | 2380995 | 79199 | loss | 33 |
| 14 | 2380996 | 2421749 | 40753 | both | 35 |
| 14 | 9389986 | 9862200 | 472214 | loss | 86 |
| 14 | 16009830 | 16074463 | 64633 | gain | 23 |
| 14 | 24670504 | 24906177 | 235673 | loss | 14 |
| 14 | 52573859 | 53488013 | 914154 | loss | 202 |
| 14 | 54540354 | 55057069 | 516715 | loss | 78 |
| 14 | 62690545 | 62728367 | 37822 | gain | 34 |
| 14 | 71748627 | 72049542 | 300915 | loss | 35 |
| 14 | 74915088 | 75672762 | 757674 | loss | 56 |
| 14 | 76391868 | 78215493 | 1823625 | loss | 17 |
| 14 | 94777468 | 94854180 | 76712 | loss | 128 |
| 14 | 102629597 | 102728645 | 99048 | loss | 154 |
| 14 | 103891750 | 104759248 | 867498 | loss | 84 |
| 14 | 105261358 | 105396350 | 134992 | loss | 114 |
| 14 | 109557530 | 109682216 | 124686 | loss | 29 |
| 14 | 136557301 | 137063119 | 505818 | loss | 24 |
| 14 | 145417993 | 145465822 | 47829 | loss | 30 |
| 14 | 145465823 | 145502096 | 36273 | both | 65 |
| 14 | 145502097 | 145620173 | 118076 | loss | 30 |
| 14 | 148961252 | 149228989 | 267737 | loss | 41 |
| 14 | 149462834 | 149682553 | 219719 | loss | 28 |
| 14 | 151265965 | 151390677 | 124712 | loss | 64 |
| 14 | 152135829 | 152836608 | 700779 | loss | 344 |
| 14 | 152836609 | 152918637 | 82028 | BOTH | 423 |
| 14 | 152918638 | 153836231 | 917593 | LOSS | 344 |
| 15 | 4970790 | 5652183 | 681393 | loss | 55 |
| 15 | 9886601 | 10424015 | 537414 | loss | 57 |
| 15 | 12320526 | 12844713 | 524187 | loss | 42 |
| 15 | 13544133 | 14225608 | 681475 | loss | 540 |
| 15 | 16828036 | 17304533 | 476497 | loss | 32 |
| 15 | 30412968 | 30537572 | 124604 | loss | 71 |
| 15 | 33268982 | 33365983 | 97001 | both | 74 |
| 15 | 33365984 | 33617369 | 251385 | loss | 61 |
| 15 | 37324732 | 37835302 | 510570 | loss | 78 |
| 15 | 48790892 | 49439076 | 648184 | loss | 338 |
| 15 | 69276645 | 70035825 | 759180 | loss | 349 |
| 15 | 100447783 | 100681987 | 234204 | loss | 49 |
| 15 | 109231500 | 109450090 | 218590 | loss | 47 |
| 15 | 110777736 | 111415595 | 637859 | loss | 17 |
| 15 | 117834922 | 127553332 | 9718410 | loss | 456 |
| 15 | 133220838 | 134508064 | 1287226 | loss | 23 |
| 15 | 134508065 | 134531546 | 23481 | both | 25 |
| 15 | 134531547 | 134919214 | 387667 | loss | 23 |
| 15 | 136160033 | 136814861 | 654828 | loss | 14 |
| 15 | 142240205 | 142372530 | 132325 | loss | 14 |
| 15 | 146682543 | 146881743 | 199200 | loss | 19 |
| 15 | 149874804 | 150299481 | 424677 | loss | 22 |
| 15 | 151295030 | 154922053 | 3627023 | loss | 430 |
| 15 | 154922054 | 154963296 | 41242 | both | 432 |
| 15 | 157523700 | 157623424 | 99724 | both | 104 |
| 15 | 157623425 | 157651972 | 28547 | loss | 45 |
| 16 | 2278462 | 3066542 | 788080 | loss | 277 |
| 16 | 11509437 | 11667410 | 157973 | loss | 57 |
| 16 | 12874893 | 13561671 | 686778 | loss | 216 |
| 16 | 15346627 | 15911006 | 564379 | loss | 25 |
| 16 | 23314834 | 23629760 | 314926 | loss | 14 |
| 16 | 27066399 | 27164752 | 98353 | loss | 46 |
| 16 | 30289787 | 30566627 | 276840 | loss | 32 |
| 16 | 33038762 | 33451789 | 413027 | loss | 200 |
| 16 | 59577747 | 59684798 | 107051 | loss | 32 |
| 16 | 84005681 | 85843097 | 1837416 | loss | 170 |
| 16 | 85843098 | 85889875 | 46777 | both | 172 |
| 16 | 85889876 | 86024383 | 134507 | loss | 170 |
| 17 | 0 | 15174 | 15174 | gain | 19 |
| 17 | 15175 | 447979 | 432804 | gain | 1148 |
| 17 | 2038218 | 2679834 | 641616 | loss | 197 |
| 17 | 3459318 | 3642846 | 183528 | loss | 24 |
| 17 | 17804821 | 18423988 | 619167 | loss | 15 |
| 17 | 64806147 | 65224808 | 418661 | loss | 17 |
| 17 | 65832052 | 66343145 | 511093 | loss | 80 |
| 17 | 68646537 | 69007554 | 361017 | loss | 144 |
| 17 | 69007555 | 69108942 | 101387 | both | 157 |
| 17 | 69108943 | 69344035 | 235092 | loss | 144 |
| 18 | 664316 | 989458 | 325142 | loss | 57 |
| 18 | 989459 | 1051157 | 61698 | both | 65 |
| 18 | 1051158 | 1255378 | 204220 | loss | 57 |
| 18 | 1255379 | 1343793 | 88414 | loss | 73 |
| 18 | 1968971 | 2290982 | 322011 | loss | 30 |
| 18 | 2354238 | 2743107 | 388869 | loss | 34 |
| 18 | 3045578 | 3306110 | 260532 | loss | 73 |
| 18 | 6303226 | 6366290 | 63064 | loss | 18 |
| 18 | 10521081 | 10661809 | 140728 | loss | 24 |
| 18 | 29295240 | 29439531 | 144291 | loss | 148 |
| 18 | 30419325 | 30638064 | 218739 | loss | 265 |
| 18 | 32189513 | 32295375 | 105862 | loss | 21 |
| 18 | 35397064 | 35558035 | 160971 | loss | 53 |
| 18 | 38488658 | 39185391 | 696733 | loss | 114 |
| 18 | 46540212 | 46557494 | 17282 | loss | 30 |
| 18 | 53566107 | 53889747 | 323640 | loss | 34 |
