# Supplementary material for: A genome-wide single nucleotide polymorphism and copy number variation analysis for number of piglets born alive
Source: BMC Genomics. 2019 Apr 27;20:321. doi: 10.1186/s12864-019-5687-0 (PMC6487013; doi:10.1186/s12864-019-5687-0)
Supplement: Supplementary file 2 — Gene ontology biological processes revealed by Panther analysis. (DOCX 40 kb) [file 12864_2019_5687_MOESM2_ESM.docx]

**Additional file 2:** Gene ontology biological processes revealed by Panther analysis.

| **Gene Ontology Biological Process** | **Genes** | |  |
| --- | --- | --- | --- |
| **Biological adhesion (GO:0022610)** | *NLGN2, NOV, ASGR2, CTNNA1* | |  |
| **Biological regulation (GO:0065007)** | *CRTC2, PELI3, LETM2, SUGT1, GLRA1, TNK1, GLP2R, PNOC, AKAP11, FZD3, BAG4, MINK1, NLGN2, ARPC2, PLPP5, INCA1, NOV, IGFBP5, TMEM88, ATP1B2, ADRB3, WNT8A, TRAPPC1, RAD9A, NMUR2, RAB13, ATP8B2, SYT12, CHRNB1, RAB1B, RHOD, MAL2, CLCF1, FGFR1, MUS81, RELA, NPR1, PFN1, EFNB3, CXCL16, KCNU1, GFRA3, TP53, CXCR2, PLSCR3* | |  |
| **Cellular component organization or biogenesis (GO:0071840)** | *KDM6B, NDEL1, TBC1D10C, KDM3B, GABARAP, CFL1, ACTB, WIPI2, MINK1, NLGN2, TBC1D5, ARPC2, TAF2, TNP1, TPM3, MYH1, LOC100625519, RAB1B, TACC1, CTNNA1, FXR2, TSGA10IP, ASH2L, RAB13, PHF23, SART1, ATP8B2, SYT12, RPS27, RHOD, FSCN1, MRPL11, PFN1, EFNB3, CORO1B, CXCL16, ANKRD28, PLSCR3, BRMS1* | |  |
| **Cellular process (GO:0009987)** |  | |  |
| Cell communication (GO:0007154) | *RAB13, LOC102165804, SYT12, CHRNB1, RHOD, MAL2, CLCF1, FGFR1, RELA, NPR1, MYOT, EFNB3, ARRB2, TNFSF11, GFRA3, TP53, CXCR2, PELI3, GLRA1, TNK1, GABARAP, GLP2R, WIPI2, ERLIN2, PNOC, AKAP11, PIK3R5, BAG4, MINK1, NLGN2, INHBA, PLPP5, PLCL2, MYH1, BMP2, NOV, IGFBP5, TMEM88, RAB1B, PPP1CA, ASGR2, CTNNA1, ADRB3, DGKH, PIK3R6, WNT8A* | |  |
| Cell cycle (GO:0007049) | *NDEL1, S100A16, S100A5, S100A3, ACTB, SCIMP, S100A9, GSG1L2, S100A12, KAT5, CDC23, PPP1CA, ESCO2, S100A14, S100A4, FGF11, CRNN, PHF23, RHOD, S100A8, MUS81, CDC25C* | |  |
| Cell proliferation (GO:0008283) | *INCA1, CLCF1* | |  |
| Cellular component movement (GO:0006928) | *NDEL1, KIF20A, AKAP11, LOC100625519, KIF13B, FXR2, PLD2, RHOD, FSCN1, EFNB3, CXCL16, KIF1C, CXCR2* | |  |
| Chromosome segregation (GO:0007059) | *NDEL1, ESCO2, PHF23, MUS81* | |  |
| Cytokinesis (GO:0000910) | *ACTB, MINK1, RHOD* | |  |
|  |  | |  |
| **Developmental process (GO:0032502)** | |  | |
| Anatomical structure morphogenesis (GO:0009653) | *LOC100625519, FAT2, FSCN1, FGFR1* | |  |
| Cell differentiation (GO:0030154) | *SPRP, MINK1, TNP1, BMP2, LOC100157968, FXR2, WNT8A, INHBA, ELF1, FGFR1, NR1D2, EFNB3, SOX15* | |  |
| Death (GO:0016265) | *MINK1, INHBA, BMP2, KAT5, NOV, PPP1CA, TNFSF11, ILF2, TP53, CXCR2* | |  |
| Ectoderm development (GO:0007398) | *UNC5D, BHLHA15, NTN1, FAT2, CREB3L4* | |  |
| Embryo development (GO:0009790) | *FAT2* | |  |
| Mesoderm development (GO:0007498) | *GLP2R, MYH1, FGF11* | |  |
| System development (GO:0048731) | *SPRP, MINK1, NLGN2, LOC100157968, TACC1,*  *FXR2, WNT8A, MAL2, FGFR1, EFNB3, SOX15, GFRA3* | |  |
| **Immune system process (GO:0002376)** | *ALOX15, TNK1, SLC11A1, ALOX12, PPP1CA, ASGR2,*  *PNKD, CLCF1, RELA, TNFSF11, PNKD, CXCL16, ILF2, CXCR2* | |  |
| **Localization (GO:0051179)** | *RHOD, GLTPD2, FSCN1, CHTOP, PITPNM1, KCTD11, ARRB2, CXCL16, CNIH2, ANKRD28, SLC25A11, KIF1C, CXCR2, PLSCR3, NDEL1, TBC1D10C, BBS1, SLC29A2, SLC36A1, GLRA1, ZDHHC24, ACTB, AP5B1, WIPI2, TBC1D5, SLC16A11, SLC27A3, DDHD2, SLC36A3, REEP2, LOC100625519, YIF1A, RAB1B, RIN1, ASGR2, FXR2, CCZ1, TRAPPC1, RAB13, PLD2, ATP8B2, CHRNB1, LOC100739863* | |  |
| **Locomotion (GO:0040011)** | *NDEL1, LOC100625519, PLD2, RHOD, FSCN1, EFNB3, CXCL16, CXCR2* | |  |
| **Metabolic process (****GO:0008152)** | *RHOD, S100A8, GLTPD2, CHTOP, FGFR1, SF3B2, MUS81, NR1D2, DPH3, LSM1, CAMTA2, AP5Z1, RELA, NPR1, BRF2, PITPNM1, PSMB6, DENND4B, DAZL, LOC100627228, PIK3R5, CDC25C, KIF1C, TP53, BRMS1, LOC102164735, NAA16, LOC100157968, ATP1B2, S100A14, MPDU1, FOSL1, S100A4, ADRB3, KIF13B, FXR2, PIK3R6, RPL15, TRAPPC1, CRNN, RAD9A, NMUR2, MARCH6, ASH2L, RAB13, PHF23, SART1, ELF1, ATP8B2, POLD4, LOC102165804, LOC100622764, RNASEK, PBK, RPS27, CARNS1, ERLIN2, ANXA6, LOC100738400, S100A9, MINK1, NLGN2, INHBA, TBC1D5, PAPOLB, TAF2, SLC27A3, S100A12, EGR1, PLPP5, ENPP2, BHLHA15, ALOX12, REEP2, BMP2, DRAP1, KAT5, INCA1, PMS2, ZNF395, RAB1B, PPP1CA, KDM6B, RPS6KB2, CRTC2, SPRP, PELI3, ENO3, SAT2, MTRF1, HSPA9, TBC1D10C, SLC29A2, EXTL3, CTSW, ALOX15, SUGT1, S100A16, KDM3B, GM2A, S100A5, KIF20A, GATAD2B, GABARAP, TMEM256, S100A3, ZDHHC24, POLR2A, LOC100620439, RCE1, WIPI2, NPAS4* | |  |
| **Multicellular organismal process (GO:0032501)** | *SPRP, GLRA1, GLP2R, TPM3, PNOC, MINK1, NLGN2, AIP, TNP1, TPM3, MYH1, NTN1, LOC100157968, TACC1, FXR2, WNT8A, NMUR2, CREB3L4, SYT12, CHRNB1, MAL2, FGFR1, NR1D2, PITPNM1, EFNB3, SOX15, ARRB2, GFRA3* | |  |
| **Reproduction (GO:0000003)** | *SCIMP, TNP1, MUS81, KCNU1, ILF2* | |  |
| **Response to stimulus (GO:0050896)** | *RAB13, POLD4, CHRNB1, RAB1B, RHOD, CLCF1, FGFR1, MUS81, NR1D2, RELA, NPR1, EFNB3, BMP2, TNFSF11, CXCL16, GFRA3, PIK3R5, IL6R, TP53, CXCR2, PELI3, HSPA9, GLRA1, GABARAP, GLP2R, CCS, WIPI2, SLC11A1, ERLIN2, PNOC, AKAP11, FZD3, MINK1, INHBA, S100A12, PLPP5, NOV, IGFBP5, TMEM88, PMS2, PPP1CA, ASGR2, ADRB3, PIK3R6, WNT8A, RAD9A, NMUR2* | |  |
| **Rhythmic process (GO:0048511)** | *NR1D2* | |  |
